# Supplementary material for: Ngn3-Positive Cells Arise from Pancreatic Duct Cells
Source: Int J Mol Sci. 2021 Aug 9;22(16):8548. doi: 10.3390/ijms22168548 (PMC8395223; doi:10.3390/ijms22168548)
Supplement: Supplementary file 1 [file ijms-22-08548-s001.zip › ijms-1337334-supplementary/Sup figure 1-3 Table.pdf]

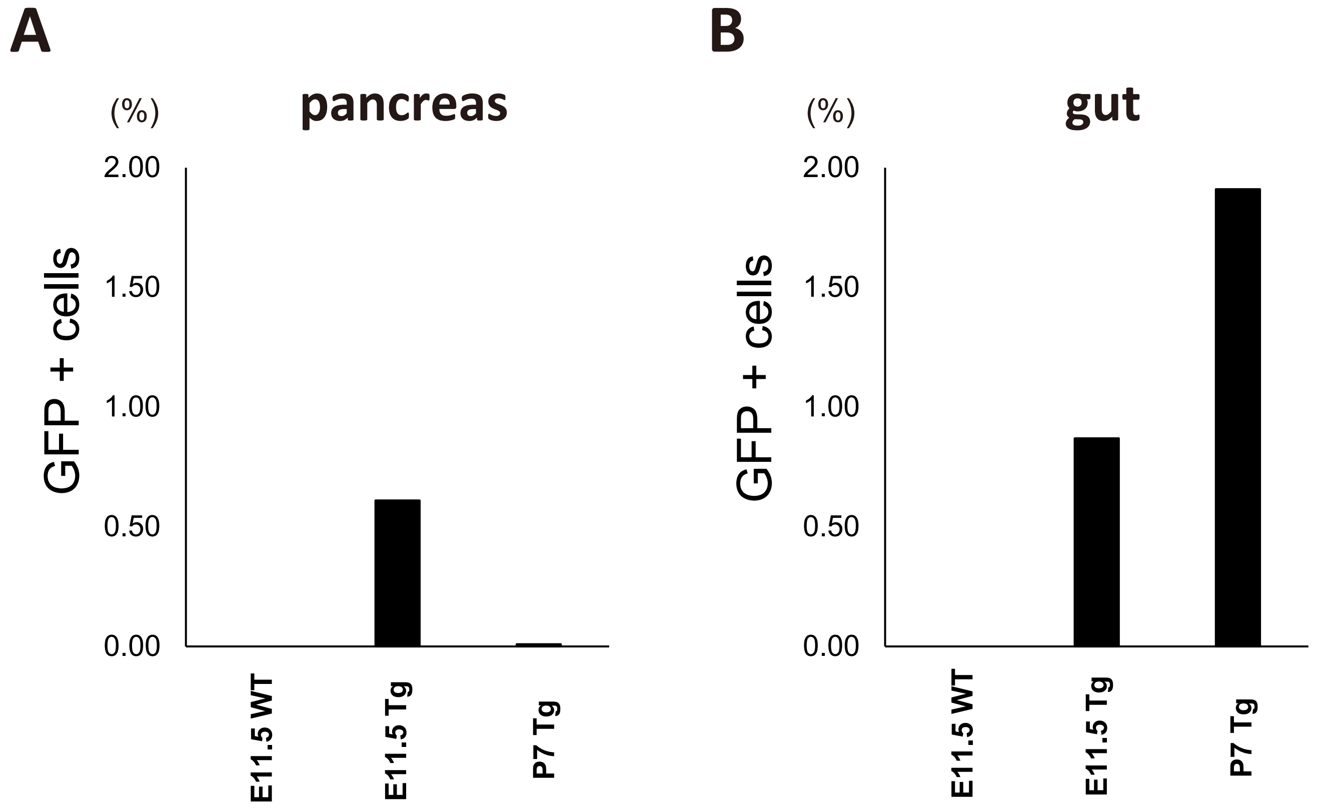


**Supplementary fig. 1**

Ngn3-positive cells appear transiently during pancreas development. Pancreas (A) and gut (B) cells dissected from Ngn3-GFP-transgenic (Tg) mouse and GFP-marked cells were counted by fluorescence activated cell sorting (FACS) system at embryonic day 11.5 (E11.5) and post-natal day 7 (P7) and the ratio of GFP-positive cells is shown by the percentage (%). GFP-positive cells from Ngn3-GFP-Tg mouse appear in the E11.5 pancreas but will almost disappear at P7. In contrast, GFP-positive cells continuously appear in the gut of P7 Ngn3-GFP-Tg mouse. E11.5 wild-type (Wt) mice were used as a negative control.

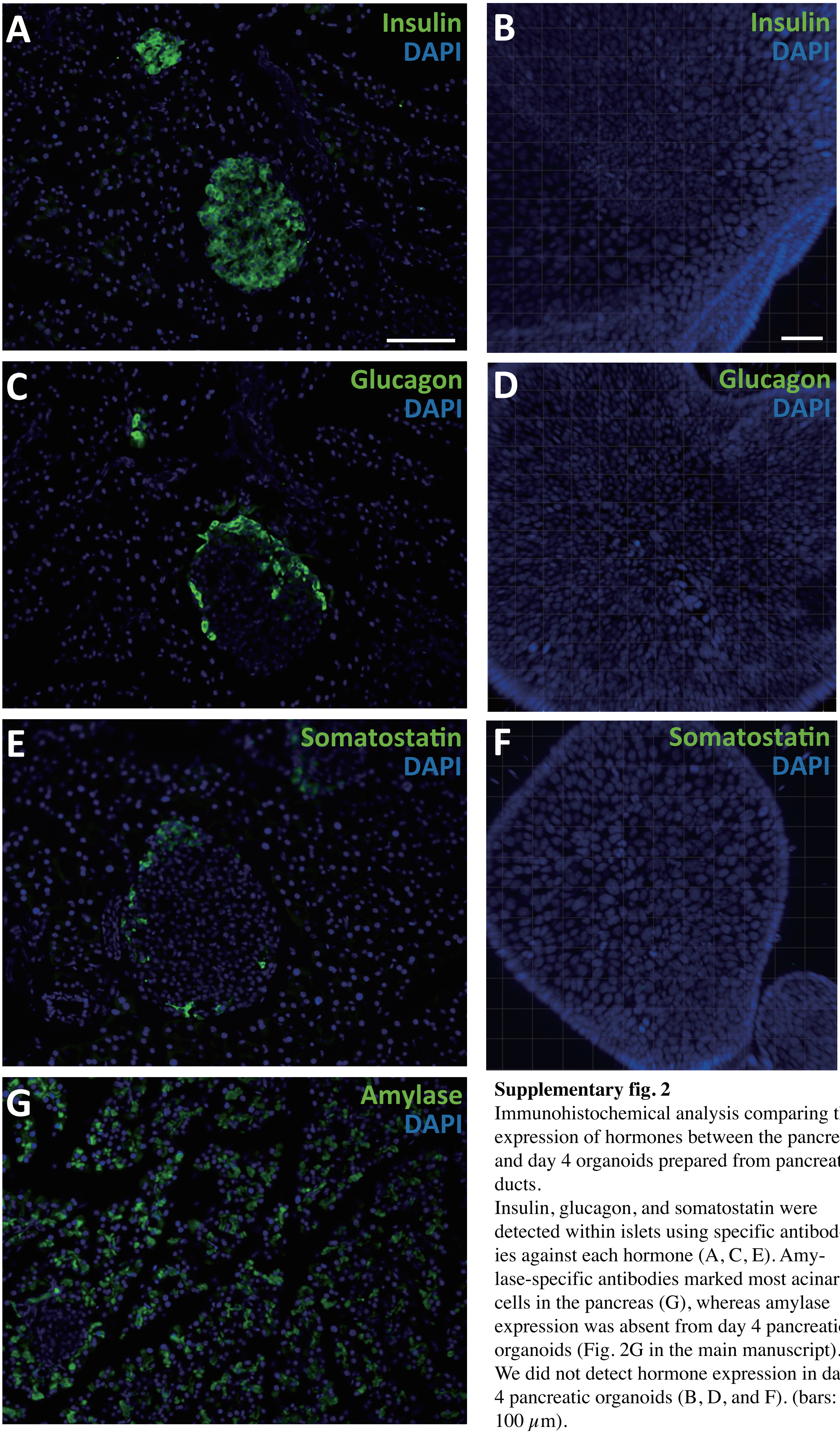


**Supplementary fig. 2**
Immunohistochemical analysis comparing the expression of hormones between the pancreas and day 4 organoids prepared from pancreatic ducts.
Insulin, glucagon, and somatostatin were detected within islets using specific antibodies against each hormone (A, C, E). Amylase-specific antibodies marked most acinar cells in the pancreas (G), whereas amylase expression was absent from day 4 pancreatic organoids (Fig. 2G in the main manuscript). We did not detect hormone expression in day 4 pancreatic organoids (B, D, and F). (bars: 100 $\mu$m).

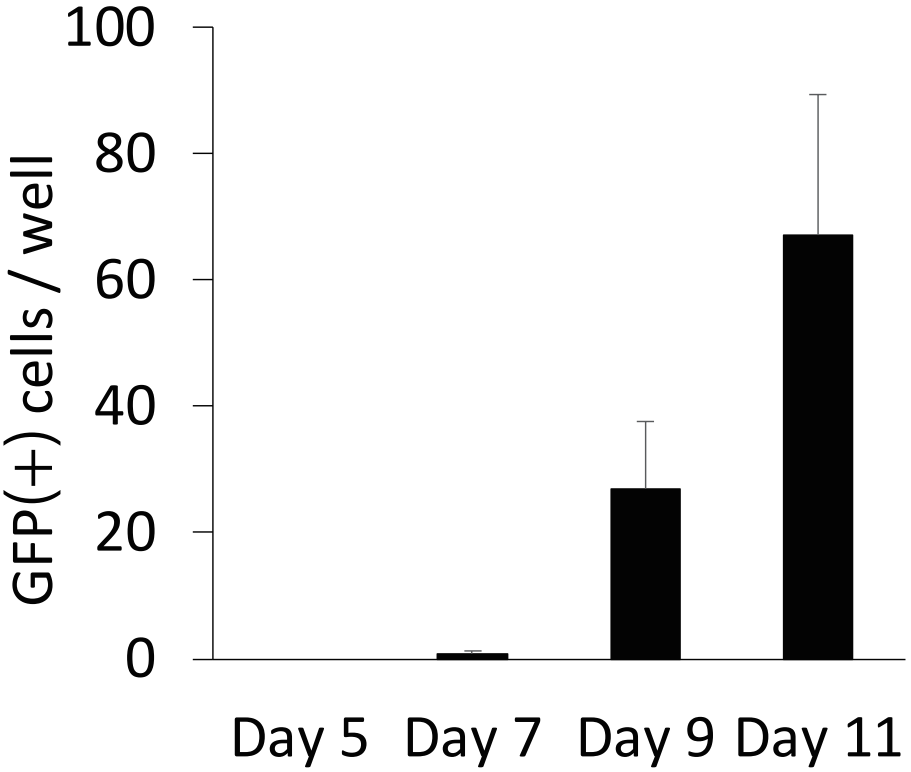


**Supplementary fig. 3**

Changes in the number of Ngn3-GFP-positive cells within pancreatic organoids generated from Ngn3-GFP mouse. GFP-positive cells per well were counted on days 5, 7, 9, and 11 of organoid culture (n = 9). GFP-positive cells were first identified on day 7, and the number of GFP-positive cells increased towards day 11.

**Table S1** Primer sequences in RT-PCR

| Gene name | Forward (5’-3’) | Reverse (3’-5’) |
|---|---|---|
| *Ngn3* | GAG TCG GGA GAA CTA GGA TG | CAG TCA CCC ACT TCT GCT TC |
| *NeuroD1* | GTC CCA GCC CAC TAC CAA TT | CGG CAC CGG AAG AGA AGA TT |
| *Isl1* | CTG CTT TTC AGC AAC TGG TCA | AGG ACT GGC TAC CAT GCT GT |
| *Gcg* | CCA CTC ACA GGG CAC ATT CA | GTC CCT GGT GGC AAG ATT AT |
| *Sst* | TCC GTC CAG TTT CTG CAG AAG | ACA GGA TGT GAA TGT CTT CC |
| *Ghrl* | GAC AAG CAG AAG AGA CAG AG | TCT TCC CAG AGG ATA TCC TG |
| *Chga* | CTG TCA GCC CTG AGT GTC TG | ATG GAA GTG GGA ACT GGA TG |
| *Trpm5* | AGT GTG ACA TGG TGG CCA TC | AGA GAA CAG TTC ACA CGA GC |
| *Gnat3* | CCA GGA ATC CAA GCC TGC TT | ACC ATG TCA TAG GCA CTT AGC |
| *Pou2f3* | GCA TCG AGA CCA ACA TAC GC | GAG ACG AAG GCC TGC TGT TG |
| *G3PDH* | ACC ACA GTC CAT GCC ATC AC | TCC ACC ACC CTG TTG CTG TA |
